# Publication Ethics in Biomedical Journals from Countries in Central and Eastern Europe

**DOI:** 10.1007/s11948-013-9431-x

**Published:** 2013-03-01

**Authors:** Mindaugas Broga, Goran Mijaljica, Marcin Waligora, Aime Keis, Ana Marusic

**Affiliations:** 1 Department of Ethical Didactics, Lithuanian University of Educational Sciences, Studentų st. 39, LT-08106 Vilnius, Lithuania; 2 Psychiatric Hospital Ugljan, Zadar, Croatia; 3 University of Split School of Medicine, Split, Croatia; 4 Department of Philosophy and Bioethics, Medical College, Jagiellonian University, Michalowskiego 12, 31-126 Krakow, Poland; 5 Department of Public Health, University of Tartu, Tartu, Estonia; 6 Department of Research in Biomedicine and Health, University of Split School of Medicine, Split, Croatia

**Keywords:** Publication ethics, Ethical criteria, Central and Eastern Europe, Research ethics

## Abstract

Publication ethics is an important aspect of both the research and publication enterprises. It is particularly important in the field of biomedical science because published data may directly affect human health. In this article, we examine publication ethics policies in biomedical journals published in Central and Eastern Europe. We were interested in possible differences between East European countries that are members of the European Union (Eastern EU) and South-East European countries (South-East Europe) that are not members of the European Union. The most common ethical issues addressed by all journals in the region were redundant publication, peer review process, and copyright or licensing details. Image manipulation, editors’ conflicts of interest and registration of clinical trials were the least common ethical policies. Three aspects were significantly more common in journals published outside the EU: statements on the endorsement of international editorial standards, contributorship policy, and image manipulation. On the other hand, copyright or licensing information were more prevalent in journals published in the Eastern EU. The existence of significant differences among biomedical journals’ ethical policies calls for further research and active measures to harmonize policies across journals.

## Background

In this article, publication ethics is understood as “a set of principles and the rules derived from them (some of the rules unwritten) that describe the proper behavior of authors, editors, reviewers, publishers, and academic and research institutions according to today’s standards” (Caelleigh 2003). Publication ethics is an emerging topic in the scientific and publishing community. Many professional organizations have promulgated ethics guidelines for publications (Bošnjak and Marušic 2012). Editors and publishers’ organizations, such as the Committee on Publication Ethics (COPE) (COPE 2012), have been established specifically to address ethical issues emerging in the publishing process (COPE 2009).

Well–publicized scandals involving scientific misconduct (Kim and Park 2012; Sokal 1996) may drive editors, publishers and ethicists to continually improve publication ethics policies. On the other hand, surveys have shown a lack of concern by editors for such issues and a common belief that misconduct occurs only rarely in their journals (Wager et al. 2009). Other surveys have demonstrated great variability in journals’ conflict of interest forms, as well as differences in practices in asking for conflict of interest declarations from authors, reviewers and editors (Cooper et al. 2006). Although scientific journals have an important role in protecting research integrity, they can only deal with the publication end of the research process; other stakeholders in the research enterprise have an even more important role in fostering the responsible conduct of research (Marušić and Marušić 2006; Marušić et al. 2007), including training in research and publication ethics (Kim et al. 2008).

Publication ethics in the field of biomedical science is particularly important because published data may directly affect human health. Publication standards in biomedicine are addressed not only by general editorial associations, such as the Committee on Publication Ethics (COPE), but also by specialized medical editorial organizations, such as the World Association of Medical Editors (WAME) and the International Committee of Medical Journal Editors (ICMJE). The biomedical research community also has a strong research activity in this field, best illustrated by the Peer Review Congress organized by JAMA and the BMJ Group. General international ethics documents devoted to biomedical research also focus on the issue of publication responsibility. Article 30 of the latest version of the WMA Declaration of Helsinki (2008) states that “Authors, editors and publishers all have ethical obligations with regard to the publication of the results of research. […]. Sources of funding, institutional affiliations and conflicts of interest should be declared in the publication”. This very influential international instrument reinforces the importance of publication ethics.

The aim of our research was to examine ethics policies of biomedical journals published in Central and Eastern Europe (CEE). We were interested in this region for several reasons. The countries in the region have evolved from similar “communist” roots and all are societies in rapid socioeconomic transition. Most have adopted international regulations, including those covering biomedical research. CEE countries constitute an attractive market for international clinical trials. However, ethical standards in the field of biomedicine are still problematic in these countries (Dranseika et al. 2011; Famenka 2011; Gefenas et al. 2010; Silis 2010; Waligora 2012).

We were interested in possible differences between East European countries that are members of the European Union (Eastern EU) and South-East European countries (South-East Europe) that are not members of the European Union. The underlying hypothesis was that the countries that have already joined the EU would have more advanced ethical requirements because of the required harmonization of their legal systems with the European Union, including the regulation of clinical trials and research on humans (Marušić 2005). We expected that biomedical journals from EU member post-communist countries would also revise their policies to reflect new regulations faster than post-communist countries outside the EU.

## Methods

We identified biomedical journals from two groups of countries: (1) East European countries within the European Union (Eastern EU): Czech Republic, Hungary, Romania, Bulgaria, Slovenia, Slovakia, Poland, Lithuania, Latvia, Estonia; and (2) South-East European countries (South-East Europe) that are not members of the European Union: Bosnia and Herzegovina, Serbia, Montenegro, Croatia, Macedonia, Albania; Greece was excluded from the study because it is a member of European Union but geographically belongs to South-East Europe.

Biomedical journals from these countries were identified using PubMed in March 2011. Search limits were set to include journals according to the country of issue, publication in English, and MEDLINE indexing. For each journal, instructions for authors were retrieved from their web pages.

The instructions for authors of these journals were then subjected to content analysis for ethics policy content (Table 1). We created a list of the most important ethical themes based on a checklist from the International Network for the Availability of Scientific Publications (INASP) and guidelines for ethical publication from ICMJE, WAME and COPE. The checklist had previously been used to assess publication practices of biomedical journals from the Eastern Mediterranean Region of the World Health Organization (Utrobičić et al. 2012).

**Table 1:** Definitions of ethical policies

Ethical policy	Definition present in guidelines for authors
Endorsement of international editorial standards	Explicit statement of the journal’s conformance with international editorial standards
Peer review process	General information on the process of evaluating manuscripts and a summary of the peer review process
Redundant publication	Policy on article submissions in print or electronic media (issues such as republications, duplicate publication, self-plagiarism, dual submission, etc.) and policy on how such occurrences are handled
Authorship policy	Policy/definition of authorship (including ethical position on ghost and gift authorship)
Contributorship policy	Clear rules on the declaration of contributorship of each co-author
Conflicts of interest	Disclosure policy of financial and personal relationships that could inappropriately influence (bias) actions
Requirements on disclosure of sources of financial support	Statement in the journal instructions about institutions and grants supporting the publication
Requirements on ethical conduct of biomedical research with human subjects	Statement of ethical biomedical research with human subjects
Requirements on ethical conduct of biomedical research with non-human subjects	Statement of ethical biomedical research with non-human subjects
Ethical review by institutional review board	Statement that submitted articles have to state whether the study received approval from a relevant ethics committee
Mandatory registration of clinical trials	Statement on mandatory registration of clinical trials
Privacy rights and confidentiality statements	Policy on proper use of personal identifiable information
Copyright or license-to-publish	Instructions regarding assignment of copyright or license-to-publish
Image manipulation	Instructions and requirements regarding processing digital images and policy for addressing image manipulation
Conflicts of editors as authors in own journals	Policy on how the journal manages research in which the editor is an author

The policies were not listed in order of importance but represented a list of publication ethics policies relevant to biomedical journals.

Results were expressed as percentages for categorical variables or mean ± SD for continuous variables. Differences were tested using χ^2^-test or Student *t* test, respectively. MedCalc version 11.5.1 was used for analysis (MedCalc, Mariakerke, Belgium).

## Results

We identified 62 journals in Eastern EU, from which we could retrieve 57 (92 %) of the instructions for authors. Out of 12 journals identified in South-East Europe, 11 (92 %) had publicly available instructions for authors.

The mean number of publication ethics policies was 5.2 ± 3.2 in journals published in EU countries and 6.6 ± 3.7 (*P* = 0.176, *t* test) in journals published outside the EU.

We identified significant differences in the presence of publication ethics policies in journals from EU and non-EU CEE countries (Table 2). The following policies were significantly more prevalent in journals published in the countries outside EU: statements on endorsement of international publishing or editorial standards (χ^2^ = 18.90, *P* < 0.001), contributorship policy (χ^2^ = 8.06, *P* = 0.005) and image manipulation (χ^2^ = 10.70, *P* = 0.001). Copyright or license-to-publish policies were more prevalent in journals published in the EU CEE countries (χ^2^ = 5.39, *P* = 0.020).

**Table 2:** Prevalence of ethics policies in Eastern EU (n = 57) and South-East Europe (n = 11)

Ethics policy	Journals from Eastern EU (%)	Journals from South-East Europe (%)	Chi square, *P*
Endorsement of international standards	10 (18 %)	9 (82 %)	18.90, *P* < 0.001
Description of process of manuscript evaluation	43 (75 %)	9 (82 %)	0.209, *P* = 0.648
Redundant publication	43 (75 %)	7 (64 %)	0.660, *P* = 0.417
Authorship policy	23 (40 %)	7 (64 %)	2.03, *P* = 0.154
Contributorship policy	9 (16 %)	6 (55 %)	8.06, *P* = 0.005
Conflicts of interest	21 (37 %)	6 (55 %)	1.21, *P* = 0.272
Requirements on disclosure of sources of financial support	16 (28 %)	4 (36 %)	0.305, *P* = 0.580
Requirements on ethical conduct of biomedical research with human subjects	26 (46 %)	8 (73 %)	2.71, *P* = 0.100
Requirements on ethical conduct of biomedical research with non-human subjects	27 (47 %)	3 (27 %)	1.51, *P* = 0.219
Ethical review by institutional review board	25 (44 %)	3 (27 %)	1.05, *P* = 0.306
Mandatory registration of clinical trials	1 (2 %)	1 (9 %)	1.74, *P* = 0.187
Privacy rights and confidentiality statements	11 (19 %)	5 (45 %)	3.51, *P* = 0.061
Copyright or license-to-publish	37 (65 %)	3 (27 %)	5.39, *P* = 0.020
Image manipulation	0 (0 %)	2 (18 %)	10.70, *P* = 0.001
Conflicts of editors as authors in own journals	1 (2 %)	1 (9 %)	1.74, *P* = 0.187

The issues most frequently addressed in Eastern EU journals were copyright and licensing (37/57 journals, 65 %), peer review process (43/57 journals, 75 %), and redundant publication (43/57 journals, 75 %). In journals from South-East Europe, the most frequent policies were on ethical conduct in clinical research (8/11 journals, 73 %), process of evaluating manuscripts and peer review process (9/11, 82 %), and statements on conformance with international editorial standards (9/11 journals, 82 %) (Table 2).

The issues least frequently addressed in Eastern EU journals were image manipulation (0/57 journals, 0 %), editors’ conflicts of interests (when editor is an author) (1/57 journals, 2 %), and registration of clinical trials (1/57 journals, 2 %). In the South-East European journals these were editors’ conflicts of interest (1/11 journals, 9 %), registration of clinical trials (1/11 journals, 9 %) and image manipulation (2/11 journals, 18 %) (Table 2).

## Discussion

Our study demonstrated significant differences in the prevalence of publication ethics policies between journals from Eastern EU and South-East Europe. Three publication ethics policies were more frequently adopted by journals published in CEE countries outside the EU: (1) statement of the journal’s conformance with international editorial standards; (2) contributorship policy, defined as “Declaration of exact contributions of each co-author, preferably in the following categories: (a) study design: (b) data collection: (c) statistical analysis: (d) literature search: (e) acquisition of funding”, and (3) image manipulation, defined as “Instructions and requirements regarding processing digital images and policy for addressing image manipulation”. The policy most often found in journals from East EU countries was copyright or license-to-publish, defined as “Instructions regarding assignment of copyright or license-to-publish”.

There may be several possible explanations for the study findings. It is possible that editors in the South-East European countries have had more editorial training or expertise, which is reflected in their journals’ policies. A recent study of Italian biomedical journals (Matarese 2008) showed that editorial leadership predicted the quality of journals, including the presence of publication ethics policies. As the public information we had available for the study did not differentiate between more professional or commercial journals and small scholarly journals, we could not relate the extent of such professionalism with the presence of ethics policies. It is also possible that journals from non-EU countries are more keen on improving their visibility in the mainstream scientific community and thus more motivated to follow developments in editorial policies and be quicker in their implementation. Furthermore, it is possible that older journals that have an established readership and visibility may pay more attention to editorial standards in general and publication ethics policies in particular. As for the difference in the prevalence of copyright or license-to-publish policies, it is possible that intellectual property rights are more respected in the EU and the journals have a legal duty to establish clear rules for publication. Another reason might be that journals in the EU countries are more concerned with the commercial aspects of publication than its ethical aspects.

In our literature search of PubMed we could not identify similar studies comparing formal ethical requirements among biomedical journals in countries undergoing socioeconomic transition in the same geographical region but under different sociopolitical influences. Therefore, it is not clear how generalizable our findings may be. Methodologically robust studies are needed to address the observed differences between countries in more detail.

Our study indicates that the least frequently addressed policies for both regions were image manipulation, conflicts arising when editors act as authors in their own journals and mandatory registration of clinical trials. A possible reason for the low prevalence of policies on image manipulation could be that most of the journals we studied were either general medical journals or journals that did not publish many articles with digital images, unlike natural science or basic biomedical research journals which originally developed the policy (Rossner and Yamada 2004). The low prevalence of the “Editor as an author” policies may be related to a lack of concern on the part of editors about their own possible conflicts of interest. Editors are usually the authors of a journals’ instructions but appear to apply them only to other authors and not to themselves. Other studies have also demonstrated that journals had an unequal application of conflict of interest policies, with authors being required to follow stricter policies than journal editors or reviewers (Cooper et al. 2006). Also, editors often do not perceive ethical issues as a relevant problem or important for their work (Wager et al. 2009).

Most of the journals in our study did not have policies on mandatory trial registration. As a public EU Clinical Trials Register (https://www.clinicaltrialsregister.eu/) was launched only in 2011, it may have not been perceived as relevant by biomedical editors in Europe. Also, most of the countries in our sample did not have local trial registries established at the time of the study, so the editors may not have been aware of the registration policy (De Angelis et al. 2004).

Our study has several limitations. Firstly, the search limits were set to include journals according to the country of publication, journals published in English and journals currently indexed in MEDLINE. There are currently many more journals in the regions which are not indexed in MEDLINE, thus our results may not be generalizable to all journals in the studied regions. Secondly, our analysis was focused on formal ethical requirements as stated in the journal instructions. It is well understood that endorsement of ethical requirements in the instructions to authors does not necessarily mean compliance with them in practice (Meerpohl et al. 2011). Thus, further research is needed to determine practical implementation and compliance with the ethical requirements in the region. Finally, the study groups may not have been fully representative of the EU and non-EU countries, as we excluded Greece because it was a member of the EU but belongs to the South-East Europe region. The wide variety of ethics publication policies in journals from South-East Europe and East EU also raises the question of how these policies and good publication practice in general can be harmonized across the countries and journals in this region. Our study suggests that journals may not succeed in this effort alone, and that action of other stakeholders in publishing is needed, from publishers and policy makers to scholarly organizations and professional associations.

A concerted action of all stakeholders is needed in the near future if the research and academic community in this European region wants to reach and/or maintain current international publication standards. The situation is alarming because many journals, which are respected in their countries and serve as important outlets for regional research, often lack even basic publishing policies, such as requirements for ethical conduct of animal research and a clear authorship policy. These policies are critical to ensure both the ethical conduct of research and publication of the research results. The journals could take the lead by reviewing their policies and describing them publicly in their guidelines to authors. This advice is relevant not only for journals in the SEE countries but generally for all journals, as several studies showed that instructions to authors do not provide an accurate and transparent description of the publication policies (Wager 2007, Matarese 2008, Meerpohl et al. 2011). The policies also need to be available in the public domain, such as on journals’ web-sites, so that the whole research community, including the journals’ readers and authors, can stay updated about the ethical requirements for publishing research.

With editors often constrained in their ability to promote publication ethics policies to control and ensure the best quality of presentation of research results, the policy makers should play a larger role in ensuring that appropriate legal and administrative tools are in place, e.g. clear rules of action of a journal or research or educational institution when there are allegations of inappropriate publication behavior. Collaboration between journals and research institutions may be important in this respect, as recently proposed by COPE (Wager and Kleinert 2012).

Finally, authors should also be aware that misconduct and disregard of ethics in research and publishing have a detrimental impact on the reputation of science in general by misleading other scientists and wasting time and resources. They should regard publication ethics not as a simple collection of rules that need to be only formally addressed but as a central part of the research enterprise.

## Appendix

Document modified by Mindaugas Broga from a checklist from INASP (International Network for the Availability of Scientific Publications): author Pippa Smart, last updated 1/24/2013 10:31:00 AM and guidelines for ethical publication from ICMJE, WAME and COPE.

## Publication Ethics Policies: Publication Ethics in Selected Countries of Central and Eastern Europe

### How to use the document

- If the journal complies with the policies, tick the box (then count the number of words for expression of the policies in the Instructions and put the number next to the tick).
- If the journal does not comply, put a cross in the box.
- If you feel a note is required, write it in the margin.

E.g. Journal X complies with the policies [a] and expresses it in 12 words. Journal X does not comply with the policies [b].

**Table:**

[a]	A statement of the journal’s conformance with **international editorial standards**	**✓12**
[b]	General information on the **process of evaluating manuscripts**, with and a summary of the peer review process	

### General information about the journal

i. Date of analysis
ii. Journal title
iii. Country
iv. Journal establishment date
v. Impact factor, indexing in bibliographical databases
vi. Time since it acquired an impact factor
vii. Type of publisher (academic/commercial)
viii. Participation in international publication ethics organizations
ix. Number of words in Instructions to Authors

**Table:**

[!]	A clearly labeled section entitled “**Information for Authors**” (or the equivalent) should contain the following:
[a]	A statement of the journal’s conformance with **international editorial standards**
[b]	General information on the **process of evaluating manuscripts**, with a summary of the peer review process
[c]	A clear statement of expectations regarding **redundant publication** (republication, duplicate publication, self-plagiarism, dual submission, “salami publishing”, etc.).
[d]	A clear statement on an **authorship policy**. No-one should be listed as a co-author who has not made a significant contribution to the work. Authors who do not meet the criteria for authorship should be listed in an acknowledgments section. Ethical position against ghost or gift-authorship.
[e]	**Contributorship policy.** The exact contribution of each co-author, preferably in the following categories: (1)study design: (2) data collection: (3) statistical analysis: (4) literature search: (5) funds collection.
[f]	Guidance on how the journal manages **conflicts of interest** between referee and author, referee and research sponsor, author and research sponsor, etc.
[g]	**Sources of financial support**. The name of the supporting institution and grant number should be given.
[h]	A clear statement of expectations regarding **ethical conduct in clinical research**.
[i]	Statement on mandatory **ethical review by institutional review board** as a prerequisite for publishing.
[j]	Statement on **mandatory registration of clinical trials** before publishing.
[k]	A clear statement of expectations regarding **ethical conduct in animal research.**
[l]	Requirements regarding observance of the **patient’s privacy rights** and **confidentiality of medical information**
[m]	Instructions regarding assignment of **copyright** or **license-to-publish**
[n]	Requirements regarding expectations on **image manipulation**.
[o]	Guidance on how the journal manages publishing of research articles in the same journal where the **researcher is also an editor**.
